# Atlanta Academy of Medicine

**Published:** 1875-06

**Authors:** Robert Battey

**Affiliations:** Reporter


### Atlanta Academy of Medicine.

ROBERT BATTEY, M.D., REPORTER.

ATLANTA, GA., April 6, 1875.

Vice-President, Dr. V. H. Taliaferro, in the chair.

Dr. Baird read an essay upon the medical uses of electricity.

Dr. Calhoun had a case of atrophy of the optic nerves in a young man of eighteen, due to pressure, as he believes, upon the commissure of the nerves. He is using the fifth of a grain of strychnia daily. He improved up to a certain point, when the case came to a stand still. Dr. Baird was kind enough to subject the patient then to daily galvanization. The latter has been continued only for one week, and there has been a gradual but sure progress. He is very favorably impressed with the progress thus far made. It is the constant current which is used in these affections.

Dr. Armstrong reported a case, the history of which dates back about two years, and which to-day resulted in the necessity for operation, by removal of the diseased eye, on account of sympathetic irritation in the sound eye.

HISTORY.—On the 14th March, 1873, M. J. K. applied for treatment of his left eye in a painful and inflamed condition. He says that he is a machinist by profession, and has been engaged at Chattanooga, Tennessee, in the railroad shops, in that capacity; that on the 4th inst. he was engaged in work on an engine, and while striking a block used in forcing some part of the machinery, it flew back and struck him in the left eye. The blow stunned him for some hours; but on the next day he continued to work, notwithstanding there was a continuous discharge of tears, pain and intolerance of light in that eye. Being a man of iron will and of great physical endurance, he continued his occupation eight days, when he came to Atlanta for treatment.

On examination, the pupil of the left eye was found to be dilated (mydriasis) to its full capacity; nor did it respond either to light or the action of calabar bean. There was photophobia, a profuse discharge of scalding tears, and general inflammation of the ball and lids, as well as severe orbital neuralgia. He could only see large objects at a short distance very indistinctly. He was placed in a dark room and free mercurial catharsis induced. Leeches were applied to the temple and around the eye, and cloths wrung in warm water used to promote a free flow of blood. From this course the pain and inflammation were, in a measure, relieved. Cold cloths were now applied, but with the effect of producing chilly sensations; they were discontinued, and cloths wet with tepid water were again used, and found soothing to the eye and comforting to patient. On the next day, 15th, there still being some orbital pain, a blister was applied to temple and poppy fomentations ordered. The gums were touched slightly with mercury to get the specific effect of the drug.

He remained in a moderately comfortable condition until the 23d, when the pain and inflammation were slightly increased. Leeches and scarrifications of conjunctiva were tried with some relief, and a blister reapplied to temple. After this scarrifications were frequently resorted to, and almost invariably with some abatement of pain and inflammation. Cold cloths were still found incompatible with his condition, and the warm cloths and poppy fomentations were continued with advantage. At no time since the patient was first seen did his pulse, while in a horizontal position, beat faster than natural, and it frequently descended to sixty beats in the minute; but on assuming the erect posture it would generally run up to one hundred.

During the month of April there was but very slight variation in the management of the eye, and the improvement was not marked until May 5th, at which time there was a decided change for the better. The pain ceased and the inflammation was much reduced. His appetite became good and light could be borne better. On the 15th he was sufficiently improved to have an ophthalmoscopic examination of the eye made. Besides some congestion of the retinal vessels, the retina was found to be detached on the nasal side of the eye. His sight was very little improved; he was only able to see the form of large objects at a hundred or more feet distant, when they were in front or to the right; those to the left being invisible on account of detachment of retina on the nasal side of eye. He remained under observation until June 1st, when he had sufficiently recovered his general health to resume his occupation.

On the 10th of February, 1875, near two years from the date of injury, M. J. K. applied to me again to treat the same eye. He says that he has been engaged without interruption at his business since his recovery, and that his eye has never given him any trouble until eight days ago, when he felt some pain and had increased flow of tears from it. These symptoms increased daily until he presented himself for treatment. He says further that the little sight left in that eye has been lost within the last few days.

Upon examination, there is the same degree of mydriasis as existed when he first came under observation. The lens is cataractous, and there is inflammation of the ball, with severe pain in the eye and around the orbit.

Leeches, scarrifications and a blister to the temple were used as before, and with the effect of greatly modifying the pain and inflammation. Cold applications were found uncomfortable; warm fomentations, on the contrary, were very soothing. His pulse was not often over sixty beats to the minute, and he suffered from gout, nervous prostration and chilly sensations.

The condition of patient remained somewhat improved until the 21st, when pain and the flow of hot, scalding tears indicated a return of the inflammation. After scarrifying the conjunctiva and applying a blister to the temple, he expressed himself as being much more comfortable. Applications of tepid water seemed to add much to his comfort also.

He complained of inability to sleep, and after this morphine was given at bed time, with the effect of relieving all pain and bringing sound sleep. No further changes worthy of notice occurred in the symptoms until 11th March, when some pain, redness and increased lachrymation were developed in the right or sound eye. The patient was at once made acquainted with the fact that sympathetic irritation had occurred in the right eye, and the danger of it explained to him. Then the removal of the diseased and blind eye, with a view of preventing further trouble in the other, was proposed. The operation, however, was declined for the time. Antiphlogistic treatment was continued, together with daily use of morphine, until the 23d March. At this date the inflammation in the diseased eye had greatly diminished, and the sympathetic irritation of the right eye apparently gone.

The same course of treatment was continued up to the 3d of April. On the night of this day the left eye again became very painful, and sympathetic irritation in the right eye came on at the same time. Vision, however, was in no way impaired in this eye. With the hope of relieving the sympathetic trouble the operation of removing the diseased eye was again proposed and urged upon the patient, which he consented to have done the next morning.

At 10 A.M. the patient was placed fully under the influence of an anæsthetic, and the diseased eye removed. The immediate relief afforded by the operation was marked; the patient expressed at once the sense that a great burden had been rolled off him.

Dr. Calhoun, in reply to a question, said that detachment of the retina, resulting from blows, is rather common. He also reported the following case: Miss ——, of Gainesville, Georgia, had been sitting up constantly with a sick relative for several weeks, and was very much prostrated when the necessity for mental and bodily strain had passed away. On awaking on the morning after the first night of refreshing sleep, she found the vision of her right eye very defective, rapidly growing worse until she was almost completely blind. This state of things had existed about two weeks, when she consulted him in reference to her vision, on the 15th February. From external appearances there was no cause for the blindness, but upon examining the eye with the ophthalmoscope, the lower part of the retina for about a third or fourth of its entire extent was detached, and as she moved the eye about in different directions the detached membrane could be plainly seen to float up and down, showing the large retinal blood-vessels, which ran over the detached portion. By dilating the pupil with atropine, the detached membrane could be much more clearly observed, floating to and fro as the direction of the eye was changed. There was no pain and no inconvenience, except the loss of vision, the patient being merely able to distinguish the movement of the hand back and forth before the eye. Between the detached retina and the choroid there seemed to be a substance, but it could not be decided positively whether it was a secretion or cysticercus or some other body, though the most reasonable supposition was that it was the first. Patient was kept under observation for several days, and put upon an alterative and tonic treatment, such as iodide potass., iron, quinine, etc., with the view of bringing about an absorption of the secretion, if such existed, and of toning up her general health.

She returned to her home to give the treatment a sufficient trial, after which, if no good had resulted, it was determined to operate upon the eye, rupturing the detached portion of retina, giving the substance behind an opportunity to escape, and thereby enabling the retina to resume its former position.

At the end of three weeks the patient returned for examination, and upon looking into the eye with the ophthalmoscope, not the least particle of the detachment could be observed, the secretion between the retina and choroid had been entirely absorbed, and the retina had completely and thoroughly replaced itself in its normal position. The vitreous was clear, and the vision was found by the test letters to be two-fifths, and she could read Snellen's test types No. 8½. According to her statement, the sight was still rapidly improving. The success of the treatment, and the disappearance of the substance which was forcing the retina away from the choroid, demonstrated the cause of the detachment to have been a serous choroiditis, the secretion pouring out of the choroidal blood-vessels and falling down between the two membranes and detaching the retina. After the absorption of this secretion the retina resumed its former natural place. Several successful operations for the restoration of detachment of the retina have been reported, but very few cases are on record of replacement taking place almost of its own accord, nature doing most of the work which restored the parts to health.

The patient remained a few, days, improving all the while, and returned to her home. Adjourned.

ATLANTA, GA., April 13, 1875.

Vice-President, Dr. J. M. Boring, in the chair.

Dr. Battey reported the case of a lady of Meriwether county, Georgia, aged twenty-five, married nine years, three children, youngest three years old. Two years ago she observed a tumor in the abdomen rising out of the pelvis, which gradually augmented in size, until at last it became so serious a burden as to confine her to her bed. She was seen at her home in January. She was a small, slender woman, much emaciated; if divested of her tumor would likely weigh not over seventy-five or eighty pounds; a woman, however, of great energy and spirit. Her abdomen was enormously distended, so much so that she required the assistance of her husband to lift the tumor in order to change her position in bed. She complained somewhat of abdominal pain, but chiefly of the great distension, of dyspnœa, of nausea and loss of appetite. A multilocular ovarian tumor was diagnosticated, consisting of two large cysts separated by a shallow sulcus, and a few smaller cysts about the base. The uterus was enlarged and rising out of the pelvis—the menses had been absent four months, and she esteemed herself to be pregnant; there had been no quickening.

In view of the rational signs of a four months' pregnancy, coupled with the very great abdominal distension, the case did not present favorably for ovariotomy, and it was deemed best to tap the largest cyst to afford temporary relief, and in the hope that the uterus would empty itself and remove the embarrassment of pregnancy from the case, or, upon the other hand, that relief might be afforded by the tapping, repeated from time to time if need be, so as to relieve the vital organs from serious embarrassment and the pregnancy go on to full term. She was therefore tapped, on the 23d January, with the assistance of Dr. John T. Dixon, the attending physician, and thirty pounds of thick, soupy fluid, of a deep chocolate color, removed. It was impracticable to empty this cyst entirely, probably ten pounds of thick, grumous material remaining behind. The outlines of the other large cyst, which lay in the right side, were now well marked; it was not interfered with. The whole tumor, with its contents, was estimated at sixty pounds. The relief afforded the patient by the tapping was marked and gratifying.

On the 26th of February, Dr. Dixon writes: "Mrs. R. is getting along badly. I saw her to-day. She is as large as before tapping, is greatly emaciated, has troublesome cough, a great deal of nausea, stomach refuses to retain almost everything. She has almost constant discharge of water from the uterus; sometimes it comes, she says, in a gush, as though her waters were broken, and then more gradually. The labia majora are so much swollen it is painful for her to sit. She is anxious for the operation, but has fears that she is not strong enough."

In a subsequent letter Dr. Dixon writes: "I was called to Mrs. R. on the 7th March, early in the morning. When I reached her she had been delivered of a child, a good large one; it was still born, and estimated to have reached the sixth month. I found her dying, but perfectly conscious. Her remark to me, when I stepped in, was: 'Doctor, clear me as quick as possible, I am flooding.' The placenta was lying in the vagina; I removed it; there was no flooding, and the clothing did not show that there had been anything like hemorrhage. I tapped her a few minutes after death; drew off the same quantity of fluid that you did when here. It was not so thick; ran without any difficulty whatever through a smaller canula than you used. The fluid drawn was quite offensive."

What was the immediate cause of death in this case? This can never be positively known, since there was no autopsy, but the statements of Dr. Dixon lead to the rational opinion that it was due to rupture of the cyst, which had again become very much distended. What might have been the eventual result had the cyst been tapped previous to the abortion could not be safely predicted. It would have warded off the danger of rupture at least, which was the probable cause of death, and would therefore likely have prolonged life. The putrid character of the contents, though, must soon have terminated life, and even if a permanent drain had been established and the cyst daily washed out, it is not likely that she could have survived the resulting inflammation of the cyst itself. Ought the tumor to have been removed on the 23d January in lieu of the tapping? It is *now* clear that it ought to have been removed, in view of the termination of the case; but *at the time* it seemed equally clear that the reduction by tapping would likely afford temporary relief, as it did very satisfactorily, and that the bulk might be kept within safe limits by repeated tappings until pregnancy was gotten rid of and opportunity afforded to somewhat recuperate the general health. It was a grievous error in the patient to refuse surgical aid until driven to it in her last and dire extremity.

Dr. Todd reported that he had observed the use of a nostrum called Collins' Antidote for Opium in as many as five cases. In no case did it accomplish any good whatever. One patient took it for two years. No benefit resulted in any case.

Dr. Battey stated that he had observed its effect in one case. The patient complained that the remedy kept her sleeping profoundly all the time, and she found it necessary to diminish the dose to one-half that directed. This nostrum is, likely, a saccharine solution of morphine and quinine, colored to please the eye. The quantity of the morphine is gradually diminished and the quinine increased in like proportion to conceal the withdrawal of the morphia. He could not see how any reputable physician could prescribe this nostrum any more than Jayne's Expectorant or Helmbold's Buchu.

Drs. Calhoun, Baird and Boring had some observation of the nostrum, and concurred in the opinion that its use could not be countenanced by reputable physicians.

Dr. Woodhull exhibited a specimen of carbazotate of ammonia and also of salicylic acid, which had been sent him for experiment. The former is on trial as a substitute for quinine as an antiperiodic, and the latter as an antiseptic and antiferment. He had tried the carbazotate of ammonia in but one case of intermittent. Three-quarter grain dose administered three times daily arrested the chill in two or three days. He had not as yet tried the salicylic acid.

Dr. Battey remarked that the carbazotate of ammonia is, as its name implies, a compound of carbazotic acid with ammonia. Carbazotic acid is a nitro-hydro-carbon, obtained by the action of nitric acid upon indigo and other organic substances. He has no experience of its medicinal use. The salt is quite bitter, reminding one of quinine. Adjourned.

---

Atlanta, Ga., April 27, 1875.

Dr. John M. Johnson, President, in the chair.

Dr. V. H. Taliaferro reported the following case:

I desire to call the attention of the Academy to a recent operation for partial rupture of the perineum; not for any special interest in the operation itself, but for remarkable sequelæ which complicated and greatly threatened the successful results.

Mrs. ———, a most estimable lady, of nervo-sanguine temperament, came under my treatment some eight or ten months since, with chronic general endometritis and anteversion of the uterus, together with a rupture of the perineum extending to the sphincter ani. The uterine disease gradually yielded to treatment, and with it a restoration of the general health and strength, when it was determined to restore the perineum, inasmuch as the anteversion still existed, though causing no inconvenience, and the patient had complained since the rupture of a sense of weakness in the parts, and involuntarily supported the perineum with the hand in the act of defecation. The patient on her back, with the knees flexed, the deformity occasioned by the rupture was seen to be very considerable, the labia gaping widely, and exposing fully one-third of the vaginal canal. The patient's general health was excellent, and she could not have been in a better condition for operation.

The bowels having been evacuated the day before by enema, the patient was put under the influence of ether by Dr. Baird, and placed in the lithotomy position. The attention of Drs. Armstrong and Baird, who assisted in the operation, was now called to the demarcation of rupture through the perineal triangle, and hence the surface to be denuded was pretty well defined. The entire cicatricial tissue thus marked out was removed with curved scissors, and followed by but little bleeding, so much so that Dr. Armstrong called attention to the very small amount of blood lost. The parts were exposed to the atmosphere and cold water sponging for some twenty or thirty minutes, and the oozing ceased. The denuded surfaces were now brought together by three deep sutures, each of which was buried in the recto-vaginal septum. The parts were accurately adjusted and the sutures secured by means of small leaden buttons, and perforated shot compressed immediately over the line of adjustment.

The operation being completed, and in every way satisfactory to the gentlemen assisting me and to myself, the patient was put to bed, with the promise that I would return at an early hour to see that she was comfortable. Returning in some three or four hours, my astonishment was great to find that the patient had been bleeding, and, as she expressed it, *flooding*. Upon my expression of surprise and doubt as to the quantity of blood as stated to have been lost, I was shown several large cloths saturated and clotted with blood. The patient was excited, faint and nauseated. Placing her before a good light, and the parts exposed, I found the vulva completely covered with blood-clot. The finger introduced into the vagina found the canal distended with clotted blood. With a stream of cold water from a Davidson syringe the parts were soon cleaned off, when I at once removed the stitches, and emptied the vagina of its accumulated clots.

Satisfying myself that the bleeding came from the wounded surface, and that the oozing then going on could not possibly prove serious (the precaution of a vaginal tampon being introduced) before my return, I went for Drs. Armstrong and Baird and instruments for replacing the stitches. In less than an hour we were again with the patient, when she was at once gotten into position as for the operation, and ether administered. Upon a careful examination of the parts, the bleeding was found to proceed from the upper vaginal border of the pared surface, and from which there was scarcely any bleeding at the time of paring. There were one or two points remote from this that bled more persistently than elsewhere, but these ceased in a reasonable time. This bleeding point was for some time exposed to the atmosphere, together with the free use of the sponge and cold water, but yet the oozing was quite free and not at all abated. It was therefore decided to ligate the bleeding vessel or vessels, and accordingly a silk ligature was thrown around them and the bleeding found to be perfectly controlled. One end of the ligature was left long and thrown up the vagina. The stitches were now replaced as before, with the exception of there being two instead of three deep sutures, these being a little more apart than before. The operation was again completed, the patient was put to bed, and a quarter of a grain of morphia administered. The urine was drawn off by catheter for five or six days; the bowels were kept constipated, and the vagina syringed with warm water once daily after the third day.

The deep sutures were removed on the fourth day, and the superficial ones on the eighth, and on the tenth day the bowels were emptied by small doses of epsom salts. The union of the parts has been complete, the perineum is perfectly restored, the ugly, gaping deformity of the labia is gone, and altogether the parts are in as natural a condition as is possible, and the patient is delighted with her improved condition.

A secondary hemorrhage, under such circumstances is, to say the least, remarkable. I have no recollection of any similar case in the history of the operation for rupture of the perineum. The only rational explanation, and which I believe is concurred in by the gentlemen present and assisting, is that the vessel or vessels at the point of the verging was prevented from bleeding immediately by the torsion from the scissors, and this, while sufficient for the time being, was insufficient to withstand the reaction in the circulation.

Dr. Battey called attention to two specimens of cystic fluid recently drawn from two cases of abdominal tumor by hypodermic syringe. The first was taken from a negro woman æt. about thirty-three. She observed, three and a half years ago, a lump of the size of a pigeon's egg in the right hypochondriac region. It was hard and freely movable in every direction, situated not very deeply under the skin.

The attention of Dr. Selman was called to it six months after its appearance. It was then a little lower than when first discovered, and about the size of a walnut. The tumor is now in the ovarian region, about the size of a fœtal head, and apparently attached to Poupart's ligament. It is not attached to the skin, but its size renders the skin covering it very tense, allowing but slight movement. It is a source of some annoyance. The pain is not great, but it interferes somewhat with flexion of the thigh, and is chafed by the clothing. Without the history of the case, should have taken it for an ovarian cyst. He considers it a cystic tumor of the abdominal wall. Regards it as a very peculiar case. The fluid is chocolate colored, and it, as well as the appearance of the tumor, reminds him of the early appearance of an ovarian tumor in a young woman in whom he made an exploratory incision a few months ago, in which the tumor bulged forward, in consequence of extensive adhesions all around, thus preventing lateral extension and forcing it to project forward.

The other fluid is from a young lady barely sixteen years of age. She observed, about a year ago, a little fulness in the lower part of the abdomen; her attention being first attracted to it from the gradually increasing tightness of her clothing. She is robust, well developed, never had any sickness. Last July the attention of Dr. Selman, who was visiting a patient in the family, was called to the enlargement of her abdomen. Her menses were regular. Some placebo was ordered. In January his attention was again called to her, and upon examination found the abdomen filled by a tumor extending from the ensiform cartilage to the pubis, and bulging anteriorly.

Dr. Battey found fluctuation clear and distinct, except at the upper and left-hand portion, where he found a hard body, resembling the spleen to the feel, and, moreover, a tympanitic sound on percussion over the spleenic region. If this hard body is the spleen, it seems to be intimately associated with the tumor. The main tumor is multilocular. The portion in the pelvis is divided into three separate portions by sulci; that part in the abdomen by three sulci. Fluctuation is not felt across these divisions. The weight of the tumor is estimated at thirty or twenty-five pounds. There is but little solid matter at its base. One cyst was tapped with the hypodermic syringe, and the fluid withdrawn was almost as clear as water, with a slight opalescence. This is very rare in multilocular tumor of the ovary.

The case is one that appeals strongly to his sympathies. The patient desires to go abroad in the community, but cannot escape the finger of scorn that is constantly pointed at her.

He proposed to her an operation for the removal of the tumor, in the face of the fact that her general health is good, and in violation of the rule amongst ovariotomists not to operate before the patient has become somewhat broken down in health. The abdominal cavity becomes, by the presence of the tumor, tolerant, and the danger from peritonitis is lessened. It is a fact in general surgery that a person worn down by caries or a semi-malignant disease, bears an operation better than one suffering from acute injury. Dangerous inflammation is risked by an operation on a person in robust health, and the moral effect of putting a vigorous person to bed is unfavorable. For these reasons it is deemed best to wait until the patient has become accustomed to carrying the burden, but it is not wise to wait until death's door is almost reached. Such is the general rule.

Dr. Baird could not understand the philosophy of deferring so grave an operation until the powers of life began to fail, particularly when we recall the admitted fact that septicæmia, and not peritonitis, is the most dreaded sequel of ovariotomy.

Dr. J. M. Johnson does not think it best to operate in full health. There should be a period of preparation. The powers of the system should be preserved, and at the same time the patient should be reduced. But reduction should not be carried too far, and the operation should not be deferred too long.

Dr. J. T. Johnson thought there was more danger from acute inflammation after operations in health; but, on the other hand, the risk of septic poisoning is increased when the vital powers are weakened.

Dr. Battey said it was true that the great danger in ovariotomy is not peritonitis from incision of the peritoneum, from exposure to the air, or from sponging its surface, but from septicæmia. Experience shows that those persons who have carried the tumor for a length of time give the greatest percentage of recoveries. It was reasoned, *a priori*, in the early history of this operation, that the earlier the tumor was removed the better for the patient. It is smaller, the adhesions are less, the stamina of the patient is better, and the power of resistance is not impaired. But experience does not verify this argument. If the cyst in the second case was not multilocular, and if the fluid contained no albumen, he would think it simply a cystic tumor of the broad ligament, and not of the ovary. In that event a simple tapping would suffice for the cure, and ovariotomy would not be justifiable. Adjourned.

---

Atlanta, Ga., May 4, 1875.

Dr. J. M. Johnson, President, presiding.

Dr. Calhoun presented an eye removed by eneucleation from a man æt. about twenty-eight. Was struck in the left eye, about last Christmas, by a small piece of wood, that punctured the coats at the corneo-sclerotic junction, allowing the humors to escape. The eye became sunken, and vision was lost. Six weeks after the accident he experienced pain in the right eye. The pain corresponded in its location exactly to the seat of the wound in the other ball, and in a few hours attained a great degree of violence. On the second day vision was entirely lost, and the pain in both eyes was very intense. The ordinary treatment, cold, rest in a dark room, and cathartics, was adopted by his physician. In two weeks he was able to go about and attend to some work.

Two weeks later both eyes were again attacked by violent inflammation. The former treatment produced no effect. A consultation was had, and it was decided to cut out the cicatrix, supposing that it might be the cause of irritation by producing traction on the ciliary nerves. During the operation the humors escaped, and temporary relief followed. After a short period sympathetic inflammation again lit up. The left eye was then incised and the fluids evacuated, with improvement for a day or two, only to be followed by another relapse.

On April 29th he was brought to Atlanta for an operation. Vision was entirely gone in the left eye, which was very much contracted. In the right could barely distinguish fingers moved before his face. He presented all the symptoms of sympathetic inflammation, and consequently the removal of the wounded eye was advised, hoping thereby to relieve the pain. The left ball was removed, and relief was experienced immediately. The next day he was entirely free from pain, and left for home, since which time he has not heard from him.

Sympathetic inflammation is most apt to follow those injuries of the ball that involve the ciliary bodies. The irritation is not transmitted by the optic, but through the ciliary neves, and sympathetic inflammation is sure to follow, sooner or later, in those cases in which destruction of the eye has followed a wound of the ciliary bodies. In these cases always advises the removal of the injured eye before sympathetic inflammation shows itself in the sound eye, for its occurrence is simply a question of time.

Recently had a case in which the man was shot in the eye by an arrow when a boy. Twenty-nine years afterward sympathetic irritation set up in the other eye. The injured ball was removed, vision in the inflamed eye was improved but not restored. Had the same experience in another case, in which the inflammation occurred after a lapse of more than thirty years. The trouble in the second eye is irido-choroiditis. It almost always begins with gradual impairment of vision, and soon inflammation and suppuration of the choroid and iris follow.

By request, Dr. Calhoun briefly described the operation for for eneucleation as follows: It is very painful, and an anæsthetic should be given. An eye speculum is introduced, the conjunctiva grasped by forceps, one blade of a pair of scissors, curved on the flat, should be passed through the conjunctiva, which must be divided all around one line behind the corneo-sclerotic junction. The recti muscles are next divided, close to their insertions. The scissors are passed behind the ball and the optic nerve divided. The ball may then be taken between the thumb and fore-finger and the oblique muscles cut. When a particularly nice operation is desired, a thread may be passed as a draw-string around the circularly incised margin of the conjunctiva, and the edges brought into apposition. If the muscles are divided at their insertions, an artificial eye, fitted on the stump, has the movements of the natural eye.

Dr. J. M. Johnson reported two cases of cerebro-spinal meningitis in children aged respectively three years and one month.

The first was seen by him on the fifth day, April 26th. Had been under the care of his partner, Dr. H. V. M. Miller. The attack was sudden and violent. He became within an hour totally dumb, blind, deaf and paralyzed. When he first saw him he could move his fingers and head; has continued to improve. Is now convalescent, but unable to speak. Was treated with calomel, quinine, bromide of potassium, frictions with lead and turpentine to the entire surface of the body, and ice bags to the head and back.

The second case in the same family was attacked suddenly on April—— Symtoms violent. Anterior fontanel prominent and hard; moaning; hands clenched; back and neck rigid. Temperature of head very high: no dilatation of pupils; some redness and swelling of left eye. Abdomen hard; straining as if in the act of defecation. Ordered calomel and castor oil; warm bath for body and cold douche to head; bromide of potassium in camphor water, and eight drops of paregoric. Next day relaxed and much better. During the afternoon all the symptoms returned with increased violence and frequent convulsions. Death ensued on the sixth day. After the recurrence of the unfavorable symptoms the body was covered with dark red spots that were as black as ink during the spasms. Thinks we have no satisfactory treatment for this disease. It is generally admitted that its cause is a specific poison, and thinks that the antidote for the poison should be sought. Thinks these cases had their origin in the circumstances surrounding the patients. The lot was in a very filthy condition. The straw under the carpets had not been changed for two years. There was a case of typhoid fever in the house last year. The parents of the two children, during their sickness, suffered with headaches, stiff necks and pains in their limbs. Adjourned.

---

Atlanta, Ga., May 11, 1875.

In the absence of the President and Vice-President, Dr. W. S. Armstrong was called to the chair.

Dr. Calhoun read an essay on fistula of the cornea and its treatment.

Dr. Armstrong said: I have been very much interested in Dr. Calhoun's paper, and am glad he has chosen fistula of the cornea for his subject. It is one in which I have been greatly interested, mainly from the fact that I have occasionally been called upon to contend with the difficulties of its treatment. There are few troubles of the eye more difficult to cure, and unless they are treated successfully none are more sure to end in loss of vision.

I will mention two cases that I treated successfully by a method entirely original with myself, so far as I am aware. One of these came under treatment three years ago, and the other during the year just past. In both cases the fistulæ were situated in the cornea, very near the sclero-corneal junction, and had existed a number of months. The ordinary methods of treatment, by a firm compress to the eye, atropia, calabar bean and bruising of the fistulous track were resorted to without success. I determined finally, in order to excite a greater degree of adhesive inflammation, to make an incision as in the operation for cataract, cutting through the cornea at a right angle to the fistula; or rather, what would be more clear, perhaps, making the incision as for iridectomy, by introducing the point of the knife at a right angle to the fistula, so as to cut the fistulous track in half, and complete the operation by passing the knife directly into the anterior chamber. By this method an incision of a little more than one-fourth of the circumference of the cornea is made, and the fistulous track is so divided as to leave one portion in the anterior and one in the posterior lip of the corneal incision.

The result, so successful in these two cases, was due, no doubt, to the increased effort at reparation, brought about by the extensive corneal section, and the probable absence of complete adaptation of the fistulous openings in the anterior and posterior lips of the wound. It is scarcely necessary to mention that this method of treatment would be less adapted to a fistulous opening the farther it is removed from the sclero-corneal junction.

The recovery in both of these cases was quick and perfect, as in an ordinary operation for cataract.

Dr. Woodhull inquired if there was any trustworthy method of treating gonorrhœa that would certainly limit the attack to less than three or four weeks.

Dr. J. T. Johnson relies entirely on local treatment when the disease comes under his care. Thinks in the first stage constitutional remedies are useless. The mildest injection that will do good is the best to use. His favorite prescription is one-twelfth to one-eighth of a grain of nitrate of silver to the ounce of water. Next to this ranks sulphate of copper one-eighth to one-quarter of a grain to the ounce. Injections should be frequent, and should not be of such strength as to produce decided pain. In obstinate cases, and in gleet, has found advantage from the introduction of a metal bougie. In the later stages of the affection, if general treatment should be initiated, prefers the oil of sandal wood to other remedies.

Dr. Armstrong concurred with Dr. Johnson as to the proper mode of treating gonorrhœa. Considers it a local affection, and regards local treatment sufficient. Washes out the urethra before using the injection. Prefers a solution of nitrate of silver, one-eighth to one-quarter of a grain to the ounce. This should be used as often as every two hours until the discharge is diminished and changed in quality; then the intervals may be longer. Treats gleet by the introduction of a steel sound, full size. A discharge from the urethra is frequently kept up by a slight stricture, of which the patient is ignorant, and the gleety discharge disappears when the stricture is cured.

Dr. Taliaferro said, when a case came early under his care, that proper treatment arrested the discharge immediately, but the treatment should be continued for ten days or two weeks, until all tenderness disappears. In carrying out his plan of treatment he confines his patient to his room, and to his bed, if possible. The parts should be frequently bathed in cold water, and an injection consisting of sulphate of zinc and acetate of lead of each one and a half or two grains, and laudanum one drachm, to water one ounce, used after each act of urination, and retained in the urethra until the salt, formed by the combination between the lead and zinc, and which is not soluble in water, settles on the sides of the canal; the fluid should then be allowed to escape guttatim. At the same time he employs a saline cathartic, alkaline drinks, and oil of erigeron canadensis.

Dr. Calhoun spoke of a case of gonorrhœa he had seen in a male infant only a few days old. The penis was swollen and tender; the discharge was profuse, and possessed all the peculiarities of gonorrhœal discharge. He supposed that it was caused in the same way that ophthalmia neonatorum is produced, by contagion from the vagina of the mother. The child recovered under the use of cold applications and injections of sulphate of zinc.

Dr. W. F. Westmoreland reported a rare and interesting case. A male infant, æt. five months; entire absence of anus, and since birth has passed fæces by the urethra. For several days after birth no fæces were passed, showing that the bowel did not terminate in the bladder, but the opening formed subsequently to birth. Several attempts had been made before he saw the child to establish an outlet, but without success. The least accumulation caused obstruction, from which he had suffered much. Three weeks ago he operated. A metal bougie was passed through the urethra into the bladder, and thence into the bowel through the recto-vesical aperture, from this opening to the natural position of the anus, being about two inches. The bowel was loaded with fæcal matter and filled the whole pelvis. After cutting down upon the end of the bougie, into the bowel, a large quantity of fæces was discovered, and the incision into the gut, that before the discharge readily admitted the index finger, had so dimin-minished from the contraction of the bowel that it became necessary to enlarge the opening. This is a practical point, and was illustrated several years ago in an operation he made for the relief of a patient in whom the bowel opened into the vagina. The opening became smaller as the bowel emptied itself, and contracted so that it was necessary to extend the incision three times. The patient above referred to did well. The unnatural discharge through the urethra has ceased; a tent is kept in the wound to insure a sufficient opening, and, with proper attention, he will recover. Adjourned.

